# Editorial: Impact of public health and social measures for COVID-19 control on infectious disease epidemiology

**DOI:** 10.3389/fpubh.2024.1440084

**Published:** 2024-07-09

**Authors:** Dong Wook Kim, Hao Lei, Shenglan Xiao, Sheikh Taslim Ali, Sukhyun Ryu, Creuza Rachel Vicente

**Affiliations:** ^1^Department of Internal Medicine, Seoul National University Hospital, Seoul, Republic of Korea; ^2^School of Public Health, Zhejiang University, Hangzhou, China; ^3^School of Public Health (Shenzhen), Sun Yat-sen University, Shenzhen, China; ^4^WHO Collaborating Centre for Infectious Disease Epidemiology and Control, School of Public Health, Li Ka Shing Faculty of Medicine, The University of Hong Kong, Pokfulam, Hong Kong SAR, China; ^5^Laboratory of Data Discovery for Health Limited, Hong Kong Science and Technology Park, Hong Kong, Hong Kong SAR, China; ^6^Department of Preventive Medicine, College of Medicine, The Catholic University of Korea, Seoul, Republic of Korea; ^7^Department of Social Medicine, Universidade Federal do Espírito Santo, Vitória, Brazil

**Keywords:** SARS-CoV-2, effect, public health and social measures (PHSM), COVID-19, public health, epidemiology, social measures, infectious diseases

## Introduction

The World Health Organization declared coronavirus disease 2019 (COVID-19) a public health emergency of global concern, which prevailed from January 2020 until May 2023. Various levels of stringency, scale, and temporal characteristics of the public health and social measures (PHSMs) were adopted to mitigate or control the impact of severe acute respiratory syndrome coronavirus 2 (SARS-CoV-2) transmission globally, with possible disease outcomes for COVID-19 and other diseases. Therefore, this Research Topic aimed to explore the scientific outcomes of the impact of COVID-19 PHSMs on the control of infectious disease transmission and associated burdens.

This Research Topic reviewed articles under the following subtopics: surveillance, modeling of the impact assessment of public health interventions, population epidemiology and case management, mass gathering preparedness, and the impact of COVID-19 on other infectious diseases.

## Surveillance

Screening and testing constitute the most critical and fundamental components of surveillance to establish case definition and confirm diagnosis. Screening strategies for COVID-19 using different testing methods such as nucleic acid and antigen testing were implemented during the COVID-19 pandemic. Cost-effectiveness analysis helped determine the optimal screening strategy that maximized societal benefit and minimized costs for the cumulative number of infections, and deaths during the pandemic (Li and Zhang).

Investigations into knowledge, attitudes, practices, and acceptance help to determine other reasons behind the effects of health interventions. Therefore, limited understanding of COVID-19 prevention and control and their impact could result in ineffective precautionary behaviors among college students living in minority areas in China, who also experienced depression and stress during the pandemic (Li et al.).

Monitoring population mobility is also a prerequisite for elucidating and predicting infectious disease transmission dynamics, since it provides insights into importation risks of infections and defines effective contacts in the community. However, mobility data do not truly reflect contacts and the probability of infection upon contact; moreover, they inadequately explain transmission dynamics compared with individual social contact data. Okada et al. conducted a time series linear regression analysis exploring the association between the 7-day moving average of the night-time population in the downtown areas of three megacities in Japan and COVID-19 incidence in 2020–2022. In the time-varying regression model, the night-time population level positively affected SARS-CoV-2 transmission over 2 years. Although further validation may be necessary, public health authorities may utilize such mobility surveillance data to infer SARS-CoV-2 disease transmission in future epidemics and their control.

## Modeling the impact assessment of public health interventions

Modeling is essential in assessing the impact of PHSMs. Yi et al. used public health data released by the National Health Commission and the Shanghai Municipal Health Commission on the COVID-19 epidemic in Shanghai (January to May 2022) and the effective reproduction number to model the public health intervention's impact during the epidemic in Shanghai. The researchers found that the effective reproduction number of SARS-CoV-2 rapidly declined from 4.02 to below 0.99 after intervention adoption, and the implementation of PHSMs reduced the number of cases and epidemic wave duration compared to the counterfactual scenario of implementing 1–4 weeks later.

## Population epidemiology and case management

Understanding the population epidemiology, stratifying its risk among individual patients, and optimizing referral at the appropriate time to mitigate case fatality is imperative.

Shao et al. investigated the epidemiology of the Omicron variant in Tibet in August 2022, using retrospective data on mild or asymptomatic patients admitted to a mobile cabin hospital. The researchers demonstrated that the Omicron variant generally resulted in fewer symptoms and shorter hospital stays than wild-type SARS-CoV-2.

Keller et al. employed a German nationwide inpatient sample to analyze predisposing factors for intensive care unit (ICU) admission among patients with COVID-19. They provided insights into the risk factors associated with a high mortality rate. Furthermore, they proposed parameters for estimating the number of patients requiring ICU admission, for which ICU capacities should be considered to ensure adequate healthcare and pandemic planning.

Kaur et al. described a manually curated and validated database of long-term care facilities in Canada. The reliability of an epidemiological study depends on data quality, and database curation and validation initiatives, which are requisites for understanding infectious disease dynamics among different subgroups and populations.

## Mass gathering preparedness

Mass gatherings have become a significant public health concern owing to their super-spreading potential for SARS-CoV-2 transmission and pandemic progression. During the COVID-19 pandemic, the 2020 Olympic Games and annual Hajj were delayed and resumed step-wise, with various PHSMs implemented to control and prevent further SARS-CoV-2 transmission.

Alhussaini et al. systematically reviewed international sports mass gatherings in 2010–2022 and explored prevention strategies against COVID-19 and other infectious diseases in such events. The researchers identified risk factors for disease transmission and proposed general and specific recommendations for pre-, during-, and post-event control strategies. They presented a model representing the three stages of prevention to be embedded within community/public social responsibility, healthcare system preparedness, and public health authorities' policies/guidelines.

## Impact of COVID-19 and PHSMs on other infectious diseases

The COVID-19 pandemic and the implementation of PHSMs modified the population behavior and contact patterns, altering the transmission dynamics of other infectious diseases. A plethora of research reveals the profound impact of COVID-19 PHSMs on mitigating other respiratory infections, such as influenza and respiratory syncytial virus. However, knowledge regarding changes in the incidence of sexually transmitted diseases, such as chlamydia, is relatively limited. Chiara et al. reported changes in the monthly and yearly incidence rates of *Chlamydia* infection in South Korea by comparing the pre-pandemic (2017–2019) and peri-pandemic (2020–2022) periods, stratified by sex, age group, and region. Overall, the researchers demonstrated the changes in the trends and absolute number of *Chlamydia* infections before and after PHSMs implementation.

## Conclusions

This Research Topic discussed diverse aspects of interventions adopted during the COVID-19 pandemic, including understanding the epidemiological characteristics, the effectiveness of interventions, human behavioral factors interfering with their adherence, and suggestions for preparing and managing future health emergencies. Additionally, beyond the COVID-19, it highlights the indirect impacts of these interventions on other diseases in general. The pandemic was undeniably catastrophic, with immeasurable social and health impacts that should be adopted as a lesson for consistent public health preparation. Therefore, we anticipate that this Research Topic will contribute to this aim.

## Author contributions

DK: Investigation, Writing – original draft, Writing – review & editing. HL: Writing – original draft, Writing – review & editing. SX: Writing – original draft, Writing – review & editing. STA: Writing – original draft, Writing – review & editing. SR: Conceptualization, Funding acquisition, Methodology, Project administration, Resources, Supervision, Validation, Writing – original draft, Writing – review & editing. CV: Conceptualization, Investigation, Writing – original draft, Writing – review & editing.

